# Antibiotic resistance and prevalence of class 1 and 2 integrons in *Escherichia coli* isolated from two wastewater treatment plants, and their receiving waters (Gulf of Gdansk, Baltic Sea, Poland)

**DOI:** 10.1007/s11356-014-3474-7

**Published:** 2014-08-29

**Authors:** Ewa Kotlarska, Aneta Łuczkiewicz, Marta Pisowacka, Artur Burzyński

**Affiliations:** 1 Genetics and Marine Biotechnology Department, Institute of Oceanology of the Polish Academy of Sciences, Powstancow Warszawy 55, 81-712 Sopot, Poland; 2 Department of Water and Wastewater Technology, Faculty of Civil and Environmental Engineering, Gdansk University of Technology, Narutowicza 11/12, 80-233 Gdansk, Poland; 3 Instituto Gulbenkian de Ciência, Rua da Quinta Grande 6, 2780-156 Oeiras, Portugal

**Keywords:** *Escherichia coli*, Wastewater treatment plant, Marine outfalls, Integrons, Gene cassettes, Antibiotic resistance

## Abstract

In this study, antimicrobial-resistance patterns were analyzed in *Escherichia coli* isolates from raw (RW) and treated wastewater (TW) of two wastewater treatment plants (WWTPs), their marine outfalls (MOut), and mouth of the Vistula River (VR). Susceptibility of *E. coli* was tested against different classes of antibiotics. Isolates resistant to at least one antimicrobial agent were PCR tested for the presence of integrons. Ampicillin-resistant *E. coli* were the most frequent, followed by amoxicillin/clavulanate (up to 32 %), trimethoprim/sulfamethoxazole (up to 20 %), and fluoroquinolone (up to 15 %)-resistant isolates. Presence of class 1 and 2 integrons was detected among tested *E. coli* isolates with rate of 32.06 % (*n* = 84) and 3.05 % (*n* = 8), respectively. The presence of integrons was associated with increased frequency of resistance to fluoroquinolones, trimethoprim/sulfamethoxazole, amoxicillin/clavulanate, piperacillin/tazobactam, and presence of multidrug-resistance phenotype. Variable regions were detected in 48 class 1 and 5 class 2 integron-positive isolates. Nine different gene cassette arrays were confirmed among sequenced variable regions, with predominance of *dfrA1-aadA1*, *dfrA17-aadA5*, and *aadA1* arrays. These findings illustrate the importance of WWTPs in spreading of resistance genes in the environment and the need for inclusion of at least monitoring efforts in the regular WWTP processes.

## Introduction

Safe and economical way of wastewater disposal is an important problem requiring proper receiver-oriented management. Nowadays, wastewater treatment focuses mainly on parameters that may cause oxygen depletion and eutrophication of the receiving waters: organic matter, nitrogen, and phosphorus. Health aspects are considered only in terms of fecal contamination and evaluated only in bathing areas by monitoring fecal indicators (*Escherichia coli* and *Enterococcus* species). However, other important aspects of wastewater discharge are currently under debate. It is suspected that clinically relevant bacteria and mobile genetic elements can survive the wastewater treatment plant processes (Reinthaler et al. 2003; D’Costa et al. 2006; Łuczkiewicz et al. 2010) and be disseminated in the receiving waters (Iwane et al. 2001; Li et al. 2009; Czekalski et al. 2012). Additionally, human-associated bacteria are regarded as important vectors of gene transmission (D’Costa et al. 2006). Thus, domestic and municipal wastewater should be considered in global antibiotic resistance gene dissemination.

Mobile genetic elements play crucial role in spreading antimicrobial-resistance genes. Among them, integrons are suspected to be the most important (Stalder et al. 2012), since they are often associated with other mobile genetic elements, such as plasmids or transposons, and are detected in various environments and matrices (Rosser and Young 1999; Goldstein et al. 2001; Elsaied et al. 2007, 2011; Gillings et al. 2009; Xia et al. 2013). Integrons are widely distributed in gram-negative bacteria; however, recent studies indicate their presence also in gram-positive species (Xu et al. 2001, 2010). About 10 % of bacterial genomes that have been partially or completely sequenced harbor this genetic element (Boucher et al. 2007). The spread of antimicrobial-resistance genes by integrons was extensively investigated primarily in clinical settings (Grape et al. 2003; Pan et al. 2006; Dubois et al. 2007). Nowadays, increased interest in their environmental role is observed (Elsaied et al. 2007; Gillings et al. 2009; Elsaied et al. 2011) and also reviewed by Stalder et al. (2012). Integrons consist of integrase (*intI*) gene, a recombination site (*attI*) and one or two promoters (Recchia and Hall 1995). Based on the amino acid sequence of the *IntI* protein, five classes of integrons have been described (Cambray et al. 2010). Classes 1, 2, and 3 are the most common. Gene cassettes are captured through recombination between *attI* site of the integron and a 59-bp element (*attC* site) from the cassette. Gene cassettes associated to class 1–3 integrons often confer resistance to aminoglycoside and β-lactam antibiotics, chloramphenicol, trimetophrim, sulfonamides, spectinomycin and others (Partridge et al. 2009; Moura et al. 2009). They can also carry other adaptive genes associated with environmental stresses or open reading frames, coding hypothetical proteins of unknown function (Elsaied et al. 2007, 2011).

The objective of this study was to investigate antibiotic-resistance profiles in *E. coli* isolated from two local wastewater treatment plants (raw and treated wastewater samples), their marine outfalls located in the Gulf of Gdansk, the Baltic Sea (Poland), and from major tributary of the Baltic Sea—the Vistula River. In order to evaluate the role of the studied wastewater effluents and tributaries in dissemination of integrons and antibiotic resistance genes in anthropogenically impacted part of the Gulf of Gdansk, prevalence of class 1 and 2 integrons among *E. coli* isolates resistant to at least one antimicrobial agent was analyzed. The association between resistance or multiresistance to tested antimicrobials and presence of integrons in *E. coli* isolates was also studied. To assess the diversity of gene cassettes in integron-positive isolates, 35 selected amplicons representing variable region of integrons were sequenced and annotated.

## Materials and methods

### Sampling sites and samples collection

**Fig. 1:**
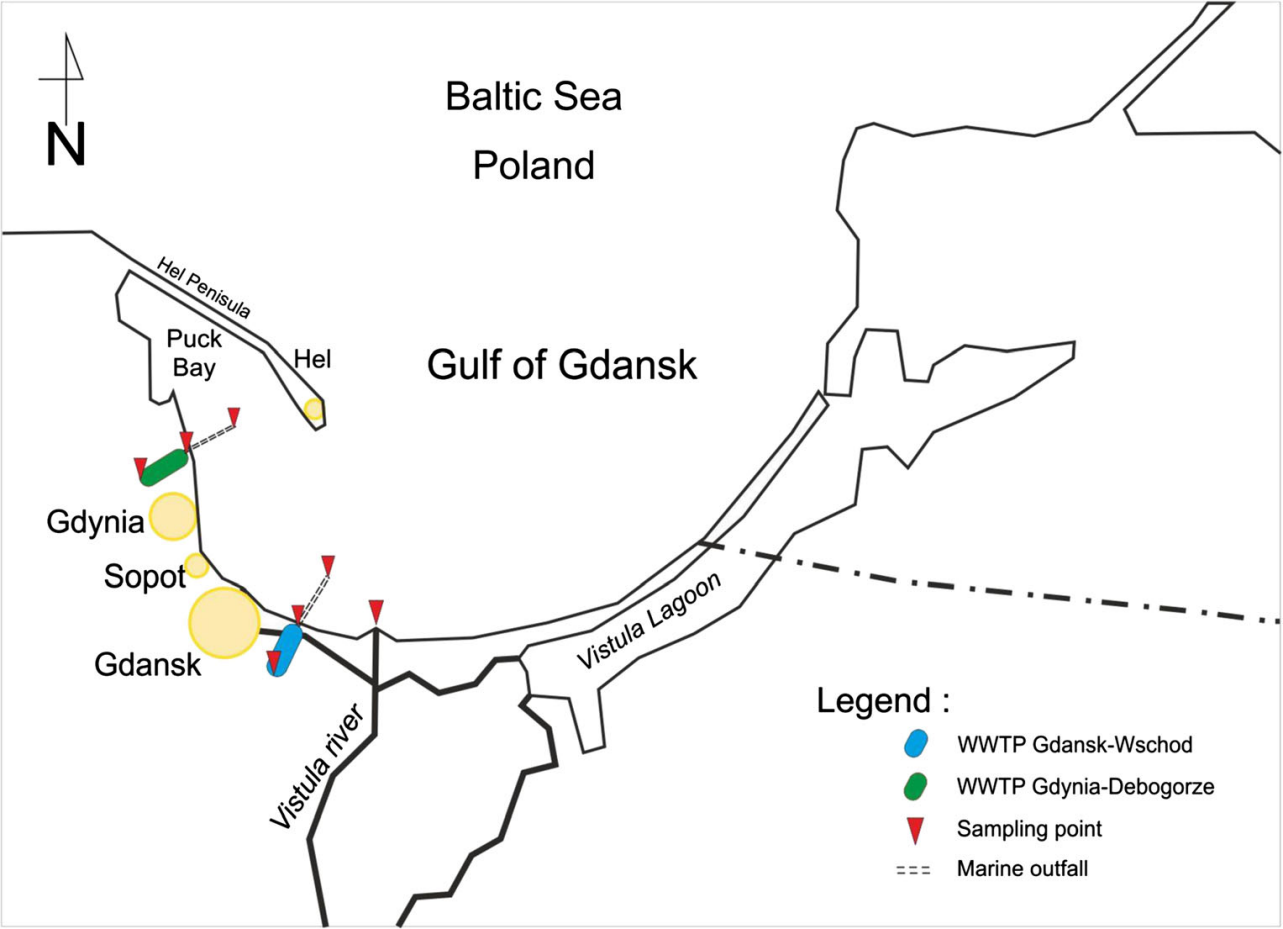
Sampling sites: raw (WRW) and treated (WTW) wastewater, as well as marine outfall (WMOut) of WWTP Gdansk–Wschod; raw (DRW) and treated (DTW) wastewater, as well as marine outfall (DMOut) of WWTP Gdynia–Debogorze. *VR* mouth of the Vistula River

Altogether, 36 samples were analyzed. Samples of raw and treated wastewater (RW and TW, respectively) were taken from two local wastewater treatment plants (WWTPs) and from their marine outfalls (MOut) (Fig. 1). In both cases, treated wastewater is discharged by submarine collectors, about 2.5 km long and equipped with diffuser systems. The plants mainly treat municipal wastewater. Industrial wastewater and non-disinfected hospital wastewater consist about 10 and 0.2 % of their daily inflow, respectively.

### WWTP “Gdansk–Wschod”

WWTP Gdansk–Wschod works in a modified University of Cape Town (UCT) type system, with integrated effective removal of nitrogen, phosphorus, and carbon in anaerobic/anoxic/oxic zones fed with internal recycles (Tchobanoglous et al. 1991). The plant serves the population of about 570,000 people (population equivalent—700,000; the average daily flow—96,000 m^3^/day). Marine outfall has been operated since 2001. The flow-proportioned composite samples of raw (WRW) and treated wastewater (WTW) were taken from February to December 2011 (six samples of each type of wastewater were collected). Marine outfall (WMOut) sampling point was located in the Gulf of Gdańsk (54° 22′ 44.4″ N; 18° 52′ 40.8″ E), and marine water was taken in April, June, August, and October 2011 (four samples in total were collected).

### WWTP “Gdynia–Debogorze”

WWTP Gdynia–Debogorze treats wastewater in four-stage Bardenpho system at the biological stage of the plant, including primary and secondary anoxic reactors (Tchobanoglous et al. 1991). The average daily flow was 55,000 m^3^/day, population equivalent—440,000. Treated wastewater has been discharged there by marine outfall since 2010. Raw (DRW) and treated wastewater (DTW) were taken as flow proportioned samples every 2 months from February to December 2011 (six samples of each type of wastewater were collected). Marine water samples (DMOut) were collected in the Puck Bay (54° 37′ 08.4″ N; 18° 33′ 28.8″ E), in April, June, August, and October 2011 (four samples in total were collected).

### Mouth of the Vistula River

Mouth of the Vistula River (VR) was sampled at the point located at 54° 22′ 58.8″ N; 18° 58′ 04.8″ E. Samples of surface water were taken once a month in April, June, August, and October 2011 (four samples in total were taken).

### Enumeration and isolation of *E. coli*

*E. coli* detection in wastewater and marine water samples was carried out according to APHA (1998) by means of membrane filtration, using membrane fecal coliform (mFC) agar (Merck) and an appropriate dilution (treated wastewater) or concentration (marine and riverine water) of collected samples. The enumeration was conducted for wastewater samples in triplicate (according to recommendations of APHA (1998) for water and wastewater samples), and for other samples, due to suspected low number of *E. coli*, in sixfold repetition. For quality control, *E. coli* ATCC 25922 strain was used. This strain forms blue and dark blue colonies on mFC agar while other organisms form gray to cream colonies. For identification and antimicrobial susceptibility tests, 5–15 blue bacterial colonies (presumptive *E. coli*) from each of the membrane filters were selected and preserved at minus 80 °C in nutrient broth (beef extract, 3 g/L, peptone 5 g/L; Becton, Dickinson and Company), supplemented with 15 % glycerol.

### Identification and antimicrobial susceptibility tests

The identification and drug susceptibility of presumptive *E. coli* isolates were determined by the Phoenix Automated Microbiology System (Phoenix AMS, BD) according to the manufacturer’s instruction. The susceptibility tests, based on microdilution, were carried out against 17 antimicrobial agents: amikacin (AN), gentamicin (GM), tobramycin (NN), imipenem (IPM), meropenem (MEM), cefazolin (CZ), cefuroxime (CXM), ceftazidime (CAZ), cefotaxime (CTX), cefepime (FEP), aztreonam (ATM), ampicillin (AM), amoxicillin/clavulanate (AMC), piperacillin/tazobactam (TZP), trimethoprim/sulfamethoxazole (STX), ciprofloxacin (CIP), and levofloxacin (LVX) together with screening for extended-spectrum β-lactamases (ESBL) production. Antimicrobial susceptibility was categorized according to EUCAST (2011). All ESBL-producing isolates were confirmed using the double-disk synergy test according to EUCAST (2011). The multidrug-resistance (MDR) phenotype was defined as simultaneous resistance to antimicrobial agents representing 3 or more categories, according to Magiorakos et al. (2012). *E. coli* ATCC 25922 and *Pseudomonas aeruginosa* ATCC 27853 were used as reference strains. Only the isolates confirmed as *E. coli* were further analyzed—774 isolates in total: 306 from WWTP Gdansk–Wschod (80 from WRW, 134 from WTW, and 92 from WMOut), 343 from WWTP Gdynia–Debogorze (124 from DRW, 102 from DTW, and 117 from DMOut), and 125 from the mouth of the Vistula River (VR).

### DNA extraction

Total DNA was obtained from several bacterial colonies, freshly grown on LB agar (tryptone 10 g/L, yeast extract 5 g/L, sodium chloride 10 g/L, agar 15 g/L; Becton, Dickinson and Company). Bacterial suspensions were prepared in distilled water and subsequently boiled in a water bath for 5 min. Cell debris was centrifuged at 13,000 rpm for 5 min, and the resulting supernatant was used as a template for all PCR assays.

### Identification and characterization of integrons

Isolates resistant to at least one antimicrobial agent (*n* = 262) were tested for the presence of integrons. These included 130 isolates from WWTP Gdansk–Wschod (35 from WRW, 49 from WTW, and 46 from WMOut), 103 from WWTP Gdynia–Debogorze (47 from DRW, 35 from DTW, and 21 from DMOut), and 29 from the mouth of the Vistula River (VR).

The presence of integrons was detected by PCR amplification of the integrase gene *intI1* (for class 1 integrons) and *intI2* (for class 2 integrons) using previously described primers (Kraft et al. 1986; Falbo et al. 1999; Mazel et al. 2000). Additionally, the presence of *sul1* and *qac*EΔ1 genes, which are usually associated with integrons (Stokes and Hall 1989; Paulsen et al. 1993), was also checked. In order to determine the size of the variable gene cassette regions in integron-positive isolates, we used previously described primer sets 5CS–3CS and hepF–hepR (for class 1 and class 2 variable region, respectively) (Levesque et al. 1995; White et al. 2001). All primers used are shown in Table 1. All PCR reactions were carried out in T1 thermal cycler (Biometra GmbH, Goettingen, Germany) using nucleotides and buffers purchased from A&A Biotechnology. For detection of integrase genes, *sul1* and *qac*EΔ1 genes *Taq* polymerase was used (A&A Biotechnology). The temperature profile for integrase, *sul1*, and *qac*EΔ1 genes amplification was as follows: initial denaturation (94 °C for 9 min), followed by 30 cycles of denaturation (94 °C for 30 s), annealing (30 s at temperature indicated in Table 1), and extension (72 °C for 1 min); and then a final extension (72 °C for 10 min). Selected class 1 and 2 integrase amplicons were sequenced and served as positive controls in further PCR experiments. Sequences of integrase genes from these positive controls were deposited in GenBank under accession numbers KM219981 and KM219982. *E. coli* DH5α strain served as negative control during PCR experiments.

**Table 1:** Primers used in this study

Target	Primer sequences (5′-3′)	PCR product size (bp)	PCR annealing temperature (°C)	Reference
*intI1*	Int1AF: CCT CCC GCA CGA TGA TC	280	55	Kraft et al. 1986
	Int1AR: TCC ACG CAT CGT CAG GC			
*intI1*	Int1BF: GAA GAC GGC TGC ACT GAA CG	1,201	65	Falbo et al. 1999
	Int1BR: AAA ACC GCC ACT GCG CCG TTA			
*intI2*	Int2AF: TTA TTG CTG GGA TTA GGC	233	50	Goldstein et al. 2001
	Int2AR: ACG GCT ACC CTC TGT TAT C			
*intI2*	Int2BF: GTA GCA AAC GAG TGA CGA AAT G	788	65	Mazel et al. 2000
	Int2BR: CAC GGA TAT GCG ACA AAA AGG T			
Class 1 integron variable region	5CS: GGC ATC CAA GCA GCA AG	Variable	63	Levesque et al. 1995
	3CS: AAG CAG ACT TGA CCT GA			
Class 2 integron variable region	hepF: CGG GAT CCC GGA CGG CAT GCA CGA TTT GTA	Variable	63	White et al. 2001
	hepR: GAT GCC ATC GCA AGT ACG AG			
*sul*1	Sul1F: ATG GTG ACG GTG TTC GGC ATT CTG A	800	55	Grape et al. 2003
	Sul1R: CTA GGC ATG ATC TAA CCC TCG GTC T			
*qac*EΔ1	qacF: ATC GCA ATA GTT GGC GAA GT	225	55	Stokes and Hall 1989
	qacR: CAA GCT TTT GCC CAT GAA GC			

In order to determine the size of variable regions of integrons, gene cassette amplifications were performed using Phusion™ polymerase (Thermo Fisher Scientific), and amplification was carried out as follows: initial denaturation (98 °C for 30 s), followed by 24 cycles of denaturation (98 °C for 10 s), annealing (63 °C for 30 s), elongation (72 °C for 6 min), and final elongation (72 °C for 10 min). PCR products were analyzed by electrophoresis on a 1 % agarose gel in 1 × TAE buffer (Sambrook and Russell 2001) and stained with ethidium bromide (0.5 μg mL^−1^), visualized under UV light, and documented using Vilber Lourmat image acquisition system. Size of the PCR products was compared with 1-kb DNA Ladder (Thermo Scientific) in Bio1D software (Vilber Lourmat, Marne-la-Vallée, France).

### Sequencing of gene cassette arrays

Selected amplicons representing various size classes of integrons were sequenced using ABI PRISM BigDye Terminator cycle sequencing with forward and reverse primers (for class 1, 5CS–3CS, and for class 2 integrons, hepF–hepR) (Macrogen, Korea). Raw sequence reads were assembled using pregap4 and gap4 programs from Staden Package (Bonfield et al. 1995). High-quality consensus sequences were extracted from the assembly and afterward analyzed. DNA sequence analysis and gene cassette array annotations were performed using BLAST algorithm against INTEGRALL database (Moura et al. 2009). Sequences were deposited in GenBank under accession numbers KJ192400–KJ192434.

### Statistical analysis

Significance of differences between antibiotic-resistance rates in all studied samples was determined using Fisher’s exact test. The frequencies of resistance to particular antimicrobials and presence of MDR phenotype in integron-positive and integron-negative isolates were compared with Fisher’s exact test. The differences in antimicrobial-resistance ranges, expressed as the number of antimicrobials or antimicrobial classes, to which the isolates were resistant, were compared with Mann–Whitney *U* test. *P* < 0.05 was considered to indicate statistical significance. Calculations were performed with Statistica 7 software (StatSoft).

## Results and discussion

### *E. coli* number

In this study, the impact posed by treated wastewater on the receiving waters was evaluated using *E. coli* isolates. The number of *E. coli* in raw wastewater (WRW, DRW) varied between 0.7 × 10^7^ and 23 × 10^7^ colony-forming unit (CFU) per 100 mL (Table 2). Both WWTPs demonstrated high efficiency in *E. coli* removal (over 99 %), although occasionally exceeding 10^5^ CFU per 100 mL in effluents (WTW, DTW). In the case of their marine outfalls (WMOut, DMOut), as well as the mouth of the Vistula River (VR), only single *E. coli* cells (<100 CFU per 100 mL) were detected, indicating that the water quality was better than the “excellent” according to the New Bathing Water Directive 2006/7/EC.

**Table 2:** Average number of *E. coli* (CFU 100 mL^−1^) in raw (WRW, DRW) and treated wastewater (WTW, DTW) as well as in marine outfalls (WMOut, DMOut), and mouth of the Vistula River (VR) (minimal and maximal number of *E. coli* in brackets)

Gdansk–Wschod			Gdynia–Debogorze			Vistula River (VR)
WRW	WTW	WMOut	DRW	DTW	DMOut	
10.7 (0.7–23) × 10^7^	2.4 (0.1–6.1) × 10^5^	43 (30–60)	8.5 (0.7–13) × 10^7^	2.1 (0.8–3.1) × 10^5^	22 (2–32)	21 (3–25)

### *E. coli* with antibiotic and multiple-antibiotic resistance

**Fig. 2:**
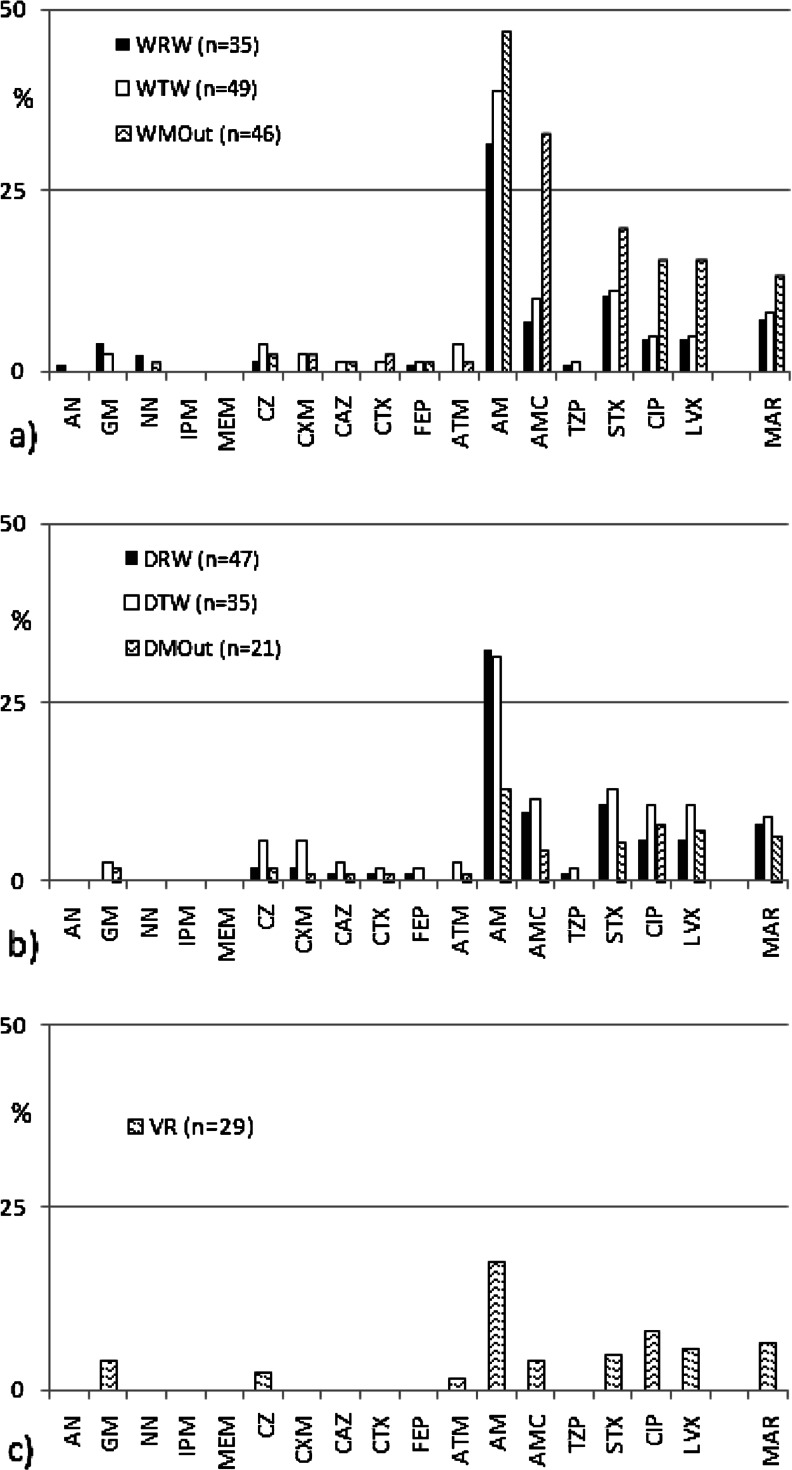
Antibiotic susceptibility of *E. coli* isolates from raw wastewater (*WRW*, *DRW*), treated wastewater (*WTW*, *DTW*), and marine outfalls (*WMOut*, *DMOut*) of **a** WWTP Gdansk–Wschod and **b** WWTP Gdynia–Debogorze, respectively, as well as from **c** the mouth of the Vistula River (*VR*)

One of the objectives of this study was to compare the antimicrobial susceptibility among *E. coli* of wastewater (WRW, WTW and DRW, DTW), marine water (WMOut and DMOut), and river mouth origin (VR). In total, 774 *E. coli* isolates were investigated. Regardless of the sampling point, high prevalence of AM-resistant *E. coli* was detected (Fig. 2). The resistance rate varied, however, in broad range, from 13 % in VR to 47 % in WMOut. The prevalence of penicillin-resistance among *E. coli* was also reported in clinical settings (ECDC 2011), as well as in different environmental compartments (Li et al. 2009). The resistance rate, however, varied significantly. In most European countries since 2007, the resistance to aminopenicillins has exceeded 40 % among *E. coli* of clinical origin (in Poland between 54 and 65 %) (ECDC 2011). The current state of knowledge suggests that the spread of antibiotic resistance is mainly due to selective pressure (ECDC/EMEA 2009; Martinez 2008; Baquero et al. 2009; Harada and Asai 2010; Andersson and Hughes 2012). Thus, these findings are not surprising—penicillins are the most often used antibacterial agents (ATC group J01) in the community (outside the hospital) in Europe (ECDC 2010). Also, broad-spectrum penicillins (J01CA) were the most consumed antibiotics in Poland—about five defined daily doses (DDD) per 1,000 inhabitants and per day, followed by combination of penicillins with β-lactamase inhibitors (J01CR)—about 3.5 DDDs per 1,000 inhabitants and per day in 2010 (ECDC 2010). In the present study, the prevalence of AM-resistant *E. coli*, followed by isolates resistant to combination of amoxicillin (penicillins) and clavulanate (β-lactamase inhibitor) (up to 32 %), seems to mirror the usage trends. Slightly lower resistance rates were noted to trimethoprim/sulfamethoxazole (5–20 %) and fluoroquinolones (5–15 %) (Fig. 2). Trimethoprim/sulfamethoxazole and fluoroquinolones are recommended as first-line drugs for uncomplicated urinary tract infections (UTIs). Recently, however, fluoroquinolones are increasingly being used instead of trimethoprim/sulfamethoxazole (Kallen et al. 2006). In consequence, selective pressure together with plasmid-mediated quinolone-resistance (PMQR) mechanisms has probably accelerated the rate of fluoroquinolone resistance spread among uropathogens (Gupta et al. 2005). In Europe, among clinical uropathogen *E. coli* strains, fluoroquinolones resistance exceeded 20 % (in Poland up to 26 % in 2010) (ECDC 2011). Quinolone and fluoroquinolone resistance is being increasingly reported around the world (Poirel et al. 2012). Principal mechanisms of resistance to those antibiotics are chromosome-encoded. However, the emergence of plasmid-mediated resistance is a major threat to public health (Mammeri et al. 2005; Poirel et al. 2012; Mokracka et al. 2012).

Among *E. coli* strains isolated from feces of mallards, herring gulls, and waterbirds on the Polish coast of the Baltic Sea, plasmid-encoded quinolone resistance associated with the *qnrS* gene was noted (Literak et al. 2010). Resistance to nalidixic acid was most frequent (together with resistance to tetracycline, followed by resistance to aminoglycosides and ampicillin) among heterotrophic marine bacteria isolated from Baltic Sea water samples (Moskot et al. 2012).

Rising trends are also observed among clinical *E. coli* resistant to third-generation cephalosporins and aminoglycosides. In this study, such resistance was reported only occasionally, while resistance to clinically relevant carbapenems (IPM and MEM) was not detected (Fig. 2). However, three isolates from raw wastewater (one from WRW and two from DRW) and two isolates from marine outfall (WMOut) produced ESBL. According to the ECDC surveillance report (2011) and numerous other reports (Pitout et al. 2005; Perez et al. 2007; Guenther et al. 2011; Liebana et al. 2013), the prevalence of ESBL-producing isolates has been on a continuous increase during the last decade. The majority of ESBLs are isolated from human clinical samples; however, they were also detected in wastewater and human-impacted environmental compartments (e.g., Galvin et al. 2010; Reinthaler et al. 2010; Hartmann et al. 2012; Chagas et al. 2011; Korzeniewska et al. 2013). ESBL-producing isolates of domestic and wild animal origin were also reported (Smet et al. 2010; Guenther et al. 2011; Tausova et al. 2012; Liebana et al. 2013). *E. coli* isolates with MDR phenotype were detected in all samples, comprising from 6 % (in VR and DMOut) to 13 % (in WMOut) of isolates. The positive selection of bacteria with resistance patterns, previously described in wastewater processes (Łuczkiewicz et al. 2010; Ferreira da Silva et al. 2007; Novo and Manaia 2010), was also observed in this study for both of the WWTPs. Resistance rate to AMC, STX, CIP, and LVX noted for *E. coli* isolated from treated wastewater (WTW and DTW) was higher than that observed in corresponding raw wastewater (WRW and DRW), but the differences were not statistically significant (in Fisher’s exact test). In the case of narrow- (CZ, CXM) and extended-spectrum (CAZ, CTX, FEP) cephalosporins, as well as TZP, the results were not evaluated due to the low number of resistant isolates. Interestingly, statistically significant rising trends (Fisher’s exact test, *P* < 0.05) were noted for AMC-, STX-, CIP-, and LVX-resistant *E. coli*, as well as for *E. coli* with MDR pattern, isolated from marine outfall WMOut and corresponding treated wastewater (WTW). Such phenomenon was not observed for treated wastewater of WWTP Gdynia–Debogorze (DTW) and its marine outfall (DMOut). Resistance rates among *E. coli* from DMOut were comparable to those detected in the mouth of the Vistula River (VR). The differences between observed local impact caused by marine outfalls may be partly explained by differences in their operation time. Treated wastewater from WWTP Gdansk–Wschod was discharged via marine outfall (WMOut) for the last 12 years, while marine outfall of WWTP Gdynia–Debogorze (DMOut) has been operating for 2 years only. However, further detailed analyses are needed to understand better the impact of resistance genes in these water ecosystems.

**Fig. 3:**
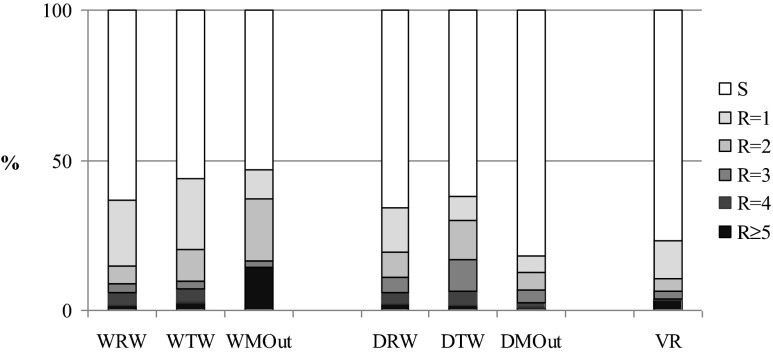
Susceptibility of *E. coli* isolates to increasing number of antimicrobial agents (*S* sensitive to all, *R1-R ≥ 5* resistant from 1 to 5 and more antimicrobial agents)

Among 37 and 34 % of *E. coli* isolated from raw wastewater samples (WRW and DRW, respectively), resistance to at least one of the antimicrobial agents was noted (Fig. 3). In both WWTP effluents, the resistance rate increased to 44 % for WTW and 38 % for DTW. In the case of marine outfalls rising, trends were observed only for WMOut (47 % of *E. coli* isolates showed resistance), while resistance rate observed in DMOut was significantly lower (18 %) and similar to that noted for mouth of the VR (23 %).

As indicated in this study, human-associated bacteria with resistance and MDR phenotypes can survive wastewater treatment processes and be disseminated in the receiving waters. Since it is believed that WWTPs represent hotspots for horizontal gene transfer (Mokracka et al. 2012; Ma et al. 2013), we decided to assess the prevalence of class 1 and 2 integrons among antimicrobial-resistant *E. coli* isolated during this study.

### Class 1 and 2 integrons in antimicrobial-resistant *E. coli*

Since integrons have been associated with antibiotic-resistance capture and dissemination, in this study, the integron presence in *E. coli* isolates resistant to at least one antimicrobial agent was tested. In general, presence of class 1 and 2 integrons was detected in *E. coli* of 32.06 % (*n* = 84) and 3.05 % (*n* = 8), respectively. Both classes of integrons were detected in one isolate (0.38 %). Other authors reported smaller amounts of integron-positive isolates in wastewater environments, but in these reports, often all *Enterobacteriaceae* or *Aeromonadaceae* isolates were taken under consideration (e.g., Koczura et al. 2012; Moura et al. 2012). Nevertheless, similar frequencies of integron-bearing *E. coli* of animal, human, and wastewater origin were noted (Cocchi et al. 2007; Vinue et al. 2008). Xia et al. (2013) reported class 2 integrons in 8 % among antimicrobial-resistant gram-negative bacteria isolated from wastewater environments in China. In our study, we did not check clonal relatedness of the isolates; thus, it could bias the results concerning prevalence of integrons. However, similar (30.8 and 1 % for class 1 and 2, respectively) (Kang et al. 2005) or even higher (85.6 and 3.6 % for class 1 and 2, respectively) (Su et al. 2006) prevalence of integrons in *E. coli* from clinical samples was recorded. In those studies, only selected strains showing the same gene cassette patterns were checked for clonal relatedness using PFGE patterns or ERIC-PCR, revealing distinct patterns; thus, little clonal relatedness between studied strains was stated (Kang et al. 2005; Su et al. 2006).

In raw wastewater, WRW and DRW, class 1 integrons were detected in 28.6 and 38.3 % of resistant *E. coli*, respectively, whereas in treated wastewater, WTW and DTW, in 26.5 and 37.1 % of isolates, respectively. In the isolates collected from marine outfalls, WMOut and DMOut, class 1 integrons were present in 29 and 37.1 % of resistant isolates respectively, while in the mouth of the Vistula River (VR) in 27.6 % of isolates.

**Fig. 4:**
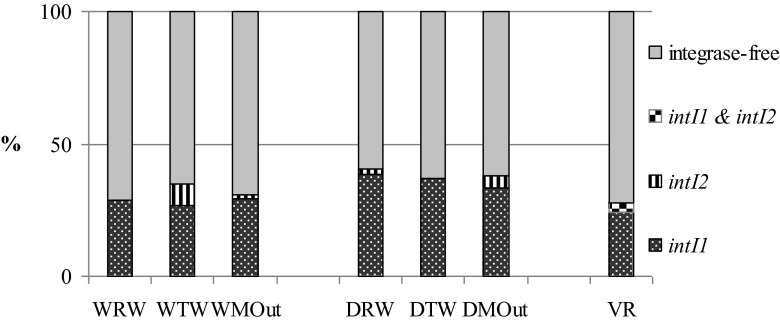
Presence of class 1 and 2 integrases in antimicrobial-resistant *E. coli* isolated from raw wastewater (*WRW* and *DRW*), treated wastewater (*WTW* and *DTW*), marine outfalls of WWTPs (*WMOut* and *DMOut*), and mouth of the Vistula River (*VR*)

Class 2 integrons were significantly less frequent. They were detected only in raw wastewater obtained from WWTP Gdynia–Debogorze (DRW) (one isolate—2.1 %), as well as in the treated wastewater of WWTP Gdansk–Wschod (WTW) (four isolates—8.2 %). In marine outfalls of both WWTPs, two isolates with class 2 integron were isolated (one isolate from each of the sampling points). Also, in the mouth of the VR, class 2 integron was detected only in one isolate. Additionally, only in this particular isolate (isolate O5 5017), integrons from both classes were present together (Fig. 4, Table 2).

Among 84 isolates positive for *intI1* gene, there were 16 isolates, in which only one pair of primers was successful in amplification of *intI1* gene (with product size of 288 bp, Table 1). These isolates were nonetheless considered as *intI1* positive because they gave positive results with primers designed to amplify variable region of class 1 integron. Among all class 2 integron-positive isolates (*n* = 8), *intI2* gene was amplified with both primers designed for this integrase gene (Table 1).

### Association between antibiotic resistance, MDR phenotype, and integrons in *E. coli* isolates

Among the integron-positive isolates, the highest resistance frequency was noted for ampicillin, amoxicillin/clavulanate, trimethoprim/sulfamethoxazole, and fluoroquinolones. Integron-negative isolates were also most frequently resistant to ampicillin, amoxicillin/clavulanate, and fluoroquinolones. Resistance to imipenem and meropenem was not observed in both groups of isolates, and none of the integron-negative isolates were resistant to amikacin. Resistance to fluoroquinolones (ciprofloxacin and levofloxacin), trimethoprim/sulfamethoxazole, amoxicillin/clavulanate, and piperacillin/tazobactam was significantly associated with the presence of integrons (Fisher’s exact test, *P* < 0.05 for piperacillin/tazobactam, *P* < 0.01 for the other abovementioned antibiotics).

Presence of integrons together with STX resistance is not surprising, since occurrence of sulfonamide resistance gene (*sul1*) is often associated with classic class 1 integrons (Paulsen et al. 1993). Correlation of other resistance with integrons is likely to the presence of numerous resistance genes in one genetic element, e.g., plasmids or transposons (Stalder et al. 2012). Similar findings were observed in heterotrophic bacteria isolated from Baltic Sea waters. In those strains, statistical analysis indicated relationships between resistance to some antibiotics (ampicillin and erythromycin, chloramphenicol and erythromycin, chloramphenicol and tetracycline, erythromycin and tetracycline), suggesting the linkage of resistance genes for antibiotics belonging to different classes (Moskot et al. 2012). No statistically significant differences were found for cephalosporins, carbapenems, aminoglycosides, and aztreonam, due to lack of or a small number of isolates resistant to those antibiotics in the study. The integron-bearing isolates were resistant to 1–13 of the tested antimicrobials belonging to 1–8 classes, and 50 % of those isolates were multidrug resistant (according to the definition proposed by Magiorakos et al. (2012)). Integron-negative isolates were resistant to 1–11 antimicrobials belonging to 1–7 classes and 17 of them (10 %) were multidrug resistant. The difference in the MDR phenotype frequency between isolates with and without integrons was statistically significant (*P* < 0.01, Fisher’s exact test). This is a common phenomenon, independent of species or origin of samples (Leverstein-van Hall et al. 2003). The differences in resistance ranges (number of antimicrobials or number of antimicrobial classes) between both groups of isolates were statistically significant (*P* < 0.001, Mann–Whitney *U* test).

### Characteristics of class 1 and 2 variable regions and gene cassette arrays

*E. coli* isolates carrying the class 1 and 2 integrons (84 and 8 isolates, respectively) were further analyzed for the presence of inserted gene cassettes in the variable region using the primer sets 5CS–3CS and hepF–hepR (Table 1), designed to amplify variable region of integrons class 1 and 2, respectively. Among all class 1 integron-positive isolates, 57.14 % (*n* = 48) possessed variable regions with size ranging from 650 to 2,600 bp. Among eight isolates bearing class 2 integrons, five isolates (62.5 %) had variable region with size ranging from 2,200 to 2,500 bp. Characteristics of those integron-bearing isolates are shown in Table 3.

**Table 3:** Characteristics of integron-bearing *E. coli* isolates isolated from WWTP “Gdansk–Wschod” and WWTP “Gdynia–Debogorze” (raw wastewater: WRW and DRW; treated wastewater: WTW and DTW), and WWTPs marine outfalls (WMOut and DMOut) and also from mouth of the Vistula River (VR)

Isolate no.	Class of integron	*sul1*	*qac*EΔ1	Antimicrobial resistance tested		Integron variable regions	
				No. of resistances	Resistance phenotype	Size of amplicon (bp)	Gene cassette array
WRW (raw wastewater from “Gdansk–Wschod” WWTP)							
GCK 27	1	+	+	2	AM STX	1,600	*dfrA17-aadA5*
GCK 140	1	+	+	1	GM	1,500	*dfrA1-aadA1*
GCK 141	1	+	+	2	AM STX	1,600	*dfrA1-aadA1*
GCK 149	1	+	+	3 (MDR)	CZ CXM AM STX	1,650	NT
GCR 150	1	+	+	3 (MDR)	AM AMC STX	1,550	NT
WTW (treated wastewater from “Gdansk–Wschod” WWTP)							
WCO 102	2	−	−	3 (MDR)	AM AMC STX	2,200	*dfrA1-sat2-aadA1* ^a^
GCO 54	2	−	−	3 (MDR)	AM AMC STX	2,300	*dfrA1-sat2-aadA1* ^a^
GCO 55	1	+	+	1	AM	1,000	*aadA1*
GCO 181	1	+	+	1	STX	1,500	*dfrA1-aadA1*
GCO 183	1	+	+	3 (MDR)	AM STX CIP LVX	1,500	*dfrA1-aadA1*
WCUV 522	1	+	+	3 (MDR)	AM STX GM NN	1,550	*dfrA17-aadA5*
GCO 175	1	+	+	2	AM STX	1,450	NT
WCO 3	1	+	+	1	AM	950	NT
GCO 164	1	+	+	4 (MDR)	AM STX CIP LVX GM	1,550	NT
GCO 187	1	+	+	1	CIP LVX	1,500	NT
GCO 188	1	+	+	1	AM	1,600	NT
WCUVO 502	1	+	+	1	AM	1,000	NT
WCUV 523	1	+	+	2	AM STX	1,500	NT
WMOut (marine outfall of “Gdansk–Wschod” WWTP)							
WWDC 701	1	−	+	2	AM STX	650	*dfrA7*
WWDC 719	1	+	+	4 (MDR)	AM AMC STX CIP LVX	1,500	*dfrA1-aadA1*
WWDC 727	2	−	−	6 (MDR, ESBL)	CZ CXM CAZ CTX FEP ATM AM AMC STX	2,500	*dfrA1-sat2-aadA1* ^a^
WWDC 743	1	+	+	2	AM STX	1,550	*dfrA17-aadA5*
WWDC 755A	1	+	+	4 (MDR)	AM AMC STX CIP LVX	1,450	NT
WWDC 755B	1	+	+	4 (MDR)	AM AMC STX CIP LVX	1,450	NT
WWDC 762A	1	+	+	4 (MDR)	AM AMC STX CIP LVX	2,000	NT
WWDC 762B	1	+	+	4 (MDR)	AM AMC STX CIP LVX	2,000	NT
WWDC 763	1	+	+	4 (MDR)	AM AMC STX CIP LVX	1,700	NT
WWDC 817	1	+	+	2	STX CIP LVX	2,000	NT
WWDC 829	1	+	+	7 (MDR, ESBL)	CZ CXM CAZ CTX FEP AM AMC STX CIP LVX NN	1,800	NT
WWDC 845A	1	+	+	4 (MDR)	AM AMC STX CIP LVX	1,700	NT
DRW (raw wastewater from “Gdynia–Debogorze” WWTP)							
BMK 231	1	+	+	3 (MDR)	AM STX CIP LVX	1,550	*dfrA1-aadA1*
BMK 236	1	+	+	2	AM STX	1,600	*dfrA17-aadA5*
BMS 512	1	+	+	3 (MDR)	AM AMC STX	1,900	*bla*_OXA30_ *-aadA1*
BMS 606	1	+	+	3 (MDR)	AM STX CIP LVX	1,550	*dfrA17-aadA5*
BMK 639	1	+	+	2	AM STX	1,800	*dfrA12-orfF-aadA2*
BMS 710	1	+	+	7 (MDR, ESBL)	CZ CXM CAZ CTX FEP ATM AM AMC STX CIP LVX	1,500	*dfrA1-aadA1*
BMS 719	1	+	+	4 (MDR)	AM AMC STX GM	1,600	*dfrA17-aadA5*
BMK 834	1	+	+	2	CIP LVX GM	1,000	*aadA1*
BMS 2009	1	+	+	2	AM STX	1,900	*dfrA12-orfF-aadA2*
BMK 2011	1	+	+	3 (MDR)	AM AMC STX	1,900	*dfrA12-orfF-aadA2*
BMS 4003	1	+	+	2	AM STX	1,500	*dfrA1-aadA1*
BMK 4016	1	+	+	2	AM STX	1,500	*dfrA1-aadA1*
BMK 26	1	+	+	3 (MDR)	AM AMC TZP	1,950	NT
DTW (treated wastewater from “Gdynia–Debogorze” WWTP)							
BMO 244	1	+	+	4 (MDR)	AM AMC STX TZP	1,500	*dfrA1-aadA1*
BMO 645	1	+	+	2	AM STX	700	*dfrA5*
BMO 843	1	+	+	1	AM	1,000	*aadA1*
BMO 3023	1	+	+	2	AM STX	1,550	*dfrA1-aadA1*
BMO 4023	1	+	+	1	AM	1,000	*aadA1*
BMO 241	1	+	+	3 (MDR)	AM STX CIP LVX	2,500	NT
BMO 5022	1	+	+	3 (MDR)	AM STX CIP LVX	2,000	NT
DCUV 14	1	+	+	2	AM STX	1,600	NT
DCUV 105	1	+	+	3 (MDR)	AM AMC STX	1,000	NT
DMOut (marine outfall of “Gdynia–Debogorze” WWTP)							
R1 3110	1	+	+	2	AM STX	1,500	*dfrA1-aadA1*
MWDC 2202	2	−	−	3 (MDR)	AM AMC STX	2,500	NT
MWDC 2203	1	−	+	1	AM	800	NT
R3 5031	1	+	+	3 (MDR)	AM STX CIP LVX	1,400	NT
VR (mouth of the Vistula River)							
O4 2092	1	+	+	1	AM	1,000	*aadA1*
O4 5012	1	+	+	1	AM	1,000	*aadA1*
O5 5017	1	+	+	2	AM STX	1,500	*dfrA1-aadA1*
	2					2,600	*estX-sat2* ^a^
UWWC 3001A	1	+	+	4 (MDR)	AM AMC STX CIP LVX	1,700	NT
UWWC 3001B	1	+	+	4 (MDR)	AM AMC STX CIP LVX	1,750	NT
O3 5008	1	+	+	3 (MDR)	AM AMC STX	1,500	NT

*AM* ampicillin, *AMC* amoxicillin/clavulanate, *ATM* aztreonam, *CZ* cefazolin, *CAZ* ceftazidime, *CTX* cefotaxime, *CXM* cefuroxime, *FEP* cefepime, *CIP* ciprofloxacin, *LVX* levofloxacin, *GM* gentamicin, *NN* tobramycin, *TZP* piperacillin/tazobactam, *STX* trimethoprim/sulfamethoxazole, *NT* not tested, *MDR* multidrug-resistance phenotype, *ESBL* extended-spectrum β-lactamase phenotype

^a^Only partial sequence was available

In some integrase-positive isolates, variable regions were not detected (with standard primers 5CS and 3CS). The absence of gene cassettes in those isolates could be possible due to the presence of a cassette array that is too large to amplify (Partridge et al. 2009). Similar findings were also noted by Ndi and Barton (2011) and Yu et al. (2003). Since occurrence of sulfonamide resistance gene (*sul1*) and quaternary ammonium compounds resistance gene (*qac*EΔ1) is often associated with classic class 1 integrons (Paulsen et al. 1993), in all *intI1*-positive isolates, presence of those genes was tested. In 25 isolates (29.76 %), *sul1* gene was not detected, and in 23 of those isolates, we failed to amplify the variable regions. In 21 isolates (25 %), *qac*EΔ1 gene was not detected, and those isolates also did not possess variable regions. Similar findings were noted for *P. aeruginosa* (Nass et al. 1998) and *E. coli* (Sáenz et al. 2004). However, there were two isolates (WWDC 701 from WMOut and MWDC 2203 from DMOut, Table 2) in which we managed to amplify variable regions, despite the lack of *sul1* gene and the presence of *qac*EΔ1 gene. There were also three isolates with mentioned above 3′ end of the integron, in which variable regions were not detected. Thus, we assume that in isolates without detectable variable regions, there are integrons with non-classic structure, probably with the *tni* region or various insertion sequences (*IS*), as mentioned by Partridge et al. (2009).

Among all integron-positive isolates, 48 isolates with class 1 integron and 5 isolates with class 2 integron contained variable regions (listed in Table 3), but only 35 selected amplicons were sequenced. Further analysis of the obtained sequences showed that the selected isolates harbored nine distinct gene cassette arrays (Table 4).

**Table 4:** Distribution of the different cassette arrays in *E. coli* isolates obtained from different sources

Type of integron and gene cassette arrays		No. of isolates	Source of isolates			
Class 1	Class 2		RW	TW	MOut	VR
*dfrA1-aadA1*	–	13	6	4	2	1
*dfrA17-aadA5*	–	6	4	1	1	–
*aadA1*	–	6	2	3	–	1
*dfrA12-orfF-aadA2*	–	3	3	–	–	–
–	*dfrA1-sat2-aadA1*	3	–	2	1	–
*dfrA7*	–	1	–	–	1	–
*dfrA5*	–	1	–	1	–	–
*bla*_OXA30_ *-aadA1*	–	1	1	–	–	–
*dfrA1-aadA1*	*estX-sat2* ^a^	1	–	–	–	1

*RW* raw wastewater, *TW* treated wastewater, *MOut* marine outfall of WWTPs, *VR* mouth of the Vistula River

^a^Only partial sequence was available

The most prevalent genes detected in variable regions of integrons were those connected with resistance to aminoglycosides (*aadA1*, *aadA2*, and *aadA5*) and trimetophrim (*dfrA1*, *dfrA5*, *dfrA7*, and *dfrA17*). Those genes were present alone, as well as in combination with each other, or other resistance genes. Genes conferring resistance to streptothricin (*estX* and *sat2*) were detected only in isolates bearing class 2 integron. One isolate, obtained from the mouth of the VR, harbored both classes of integrons, with gene cassette arrays: *dfrA1-aadA1* (class 1 integron) and *estX-sat2* (class 2 integron). Similar patterns were detected in *Aeromonas* and *Enterobacteriaceae* strains isolated from slaughterhouse WWTP (Moura et al. 2007), in *E. coli* of human and animal origin in Korea (Kang et al. 2005) and *E. coli* from Seine Estuary in France (Laroche et al. 2009). Among all sequenced variable regions of integrons, the most prevalent cassette arrays were *dfrA1-aadA1*, *dfrA17-aadA5*, and *aadA1* (Table 4).

It is interesting to find that some patterns were found only in isolates from raw wastewater, like *dfrA12-orfF-aadA2* while others only in treated wastewater or marine outfalls of WWTPs (*dfrA5* and *dfrA7*, respectively) (Table 4).

## Conclusions

Data obtained in this study indicated that wastewater treatment processes together with effective dilution of treated wastewater by marine outfall were generally sufficient to protect coastal water quality from sanitary degradation. However, human-associated bacteria, even potential pathogens and bacteria carrying antibiotic resistance genes of clinical significance, survived in wastewater and marine water conditions. Moreover, these resistant bacteria were enriched in highly diverse integrons, with nine different gene cassette arrays. Statistically significant association between resistance to fluoroquinolones, trimethoprim/sulfamethoxazole, amoxicillin/clavulanate, and piperacillin/tazobactam and the presence of integrons in *E. coli* isolates was noted. Integrons are clearly important for the development of the MDR phenotype. In conclusion, data obtained during this study indicate the potential of WWTP’s effluents in facilitating horizontal gene transfer of mobile genetic elements and MDR phenotypes in the studied area. Given the similarity of WWTP processes and municipal wastewater composition, it is likely the common problem requiring further investigation.
